# Spider Toxin Peptide-Induced NIR Gold Nanocluster Fabrication for GSH-Responsive Cancer Cell Imaging and Nuclei Translocation

**DOI:** 10.3389/fbioe.2021.780223

**Published:** 2021-11-16

**Authors:** Huaxin Tan, Sisi Liu, Yaolin He, Guofeng Cheng, Yu Zhang, Xiaojie Wei, Lidan Hu

**Affiliations:** ^1^ The Key Laboratory of Ecological Environment and Critical Human Diseases Prevention of Hunan Province Department of Education, Department of Biochemistry and Molecular Biology, School of Basic Medicine, Hengyang Medical School, University of South China, Hengyang, China; ^2^ School of Public Health, Hengyang Medical School, University of South China, Hengyang, China; ^3^ The Second Affiliated Hospital, Department of Radiotherapy, Hengyang Medical School, University of South China, Hengyang, China; ^4^ School of Pharmacy, Hengyang Medical School, University of South China, Hengyang, China

**Keywords:** NIR gold nanoclusters, spider toxin peptide, cancer cell imaging, nuclei translocation, GSH-responsive

## Abstract

Goldnanoclusters (GNCs) have become a promising nanomaterial for bioimaging because of their unique optical properties and biocompatibility. In this study, lycosin-I peptide, which possesses a highly selective anticancer activity by affecting the permeability of cancer cell membrane, was firstly modified for constructing fluorescent GNCs (LGNCs) for bioimaging of tumor cells. The obtained LGNCs exhibited strong near-infrared (NIR) fluorescence, which can be further enhanced by the peptide-induced aggregation and selectively stained three cancerous cell lines over normal cell lines with low intrinsic toxicity. After uptake by tumor cells, LGNC aggregates can be depolymerized into ultrasmall nanoclusters by high-level glutathione (GSH) and realize the nuclear targeting translocation. Collectively, our work suggests the potential of natural active biomolecules in designing NIR fluorescent GNCs for bioimaging.

## Introduction

Metal nanoclusters (MNCs) are relatively stable nanostructures composed of several to a dozen metal atoms with ultrasmall sizes (<2 nm) (Lu and Chen, 2012; Zheng et al., 2015; Jin et al., 2016; Su et al., 2020). When the size of the metal particle is similar to the Fermi wavelength of the electron, the energy level becomes discontinuous due to the quantum size effect, and the excited electron transition can produce strong fluorescence (Su et al., 2020). Compared to traditional organic fluorescent dyes and quantum dots, the size-dependent and tunable photoluminescence (PL) properties from ultraviolet (UV) to near-infrared (NIR) region make MNCs promising probes in environmental detection, molecular labeling, and biological imaging (Chang et al., 2019; Yougbare et al., 2019; Kumar et al., 2020; Schwartz-Duval et al., 2020). As the most studied MNCs, the gold nanoclusters (GNCs) have been extensively studied in the past decade especially in nanomedicine, including biosensing, bioimaging, drug delivery, and therapy, for their easy preparation, ideal photostability, and excellent biocompatibility (Su et al., 2020; Xia and Wu, 2020; Gao et al., 2021). Even so, the development of GNCs is postponed by the uncompetitive PL quantum yield (QY) and agglomeration in aqueous suspension due to the high activity of bare GNCs (You and Tseng, 2019; Wu et al., 2020).

The emerging strategy to solve this problem is combining GNCs with protective mercaptide agents, which possess high affinity with gold surfaces *via* Au–S covalent interactions (Luo et al., 2012; Yahia-Ammar et al., 2016; Yao et al., 2017; Li et al., 2021). In addition to the common monolayer protection method, more and more researchers are favoring the *in situ* synthesis method using biomolecules (DNA, proteins, peptides, etc.) as templates. Among them, thiol-containing protein and peptides have some advantages in the construction of GNCs for biological application (You and Tseng, 2019; Su et al., 2020). Firstly, the abundant hydrophilic groups on proteins and peptides are beneficial for improving the colloidal stability of conjugated GNCs. Secondly, some reductive amino acids (tryptophan, tyrosine, etc.) can reduce Au^3+^ ions to Au atoms at the appropriate pH condition, avoiding the use of strong reductants (such as NaBH4, CTAB, etc.) in GNC preparation, which provides the nanoclusters’ better biocompatibility. Thirdly, by simply changing the amino acid sequences of the templates, the atomic number, size, PL properties, and other physicochemical properties of GNCs can be quickly adjusted, which is convenient for the construction of versatile GNCs to meet different needs. Finally, yet importantly, the biological activities and functional motifs of proteins or peptides offer rich platforms for the multi-functionalization of GNCs, realizing the integration of synthesis, protection, and modification (Wang et al., 2012; He et al., 2019; Su et al., 2020). Therefore, the exploitation and utilization of novel active peptides and proteins in designs of GNCs for biomedical applications are still in great demand.

Natural toxin peptides are functional peptides with different biological activities and site specificity in the venoms of poisonous animals (including spiders, snakes, centipedes, snails, etc.). As molecular probes, they play important roles in the treatment and mechanism research of various diseases (Lüddecke et al., 2021). In our early work, lycosin-I, a toxin peptide from the *Lycosa singoriensis* spider, was found to possess an efficient and selective anticancer activity, which has great potential in the molecular design of new antitumor drugs. Lycosin-I is composed of 24 amino acid residues and contains multiple alkaline amino acid residues, which can form amphiphilic α-helix structure (Liu et al., 2012). It has been proved that lycosin-I has a potent cell-penetration ability to cancer cells. Even at a low concentration below IC_50_, it can still enter cancer cells freely and alter cell permeability at the same time (Liu et al., 2012; Tan et al., 2013). Recently, we constructed lycosin-I functionalized spherical gold nanoparticles (GNPs) and found that the peptide-modified GNPs could translocate inside tumor cells efficiently *in vitro* and *in vivo* with certain cell selectivity over normal cell (Tan et al., 2017).

**FIGURE 1:**
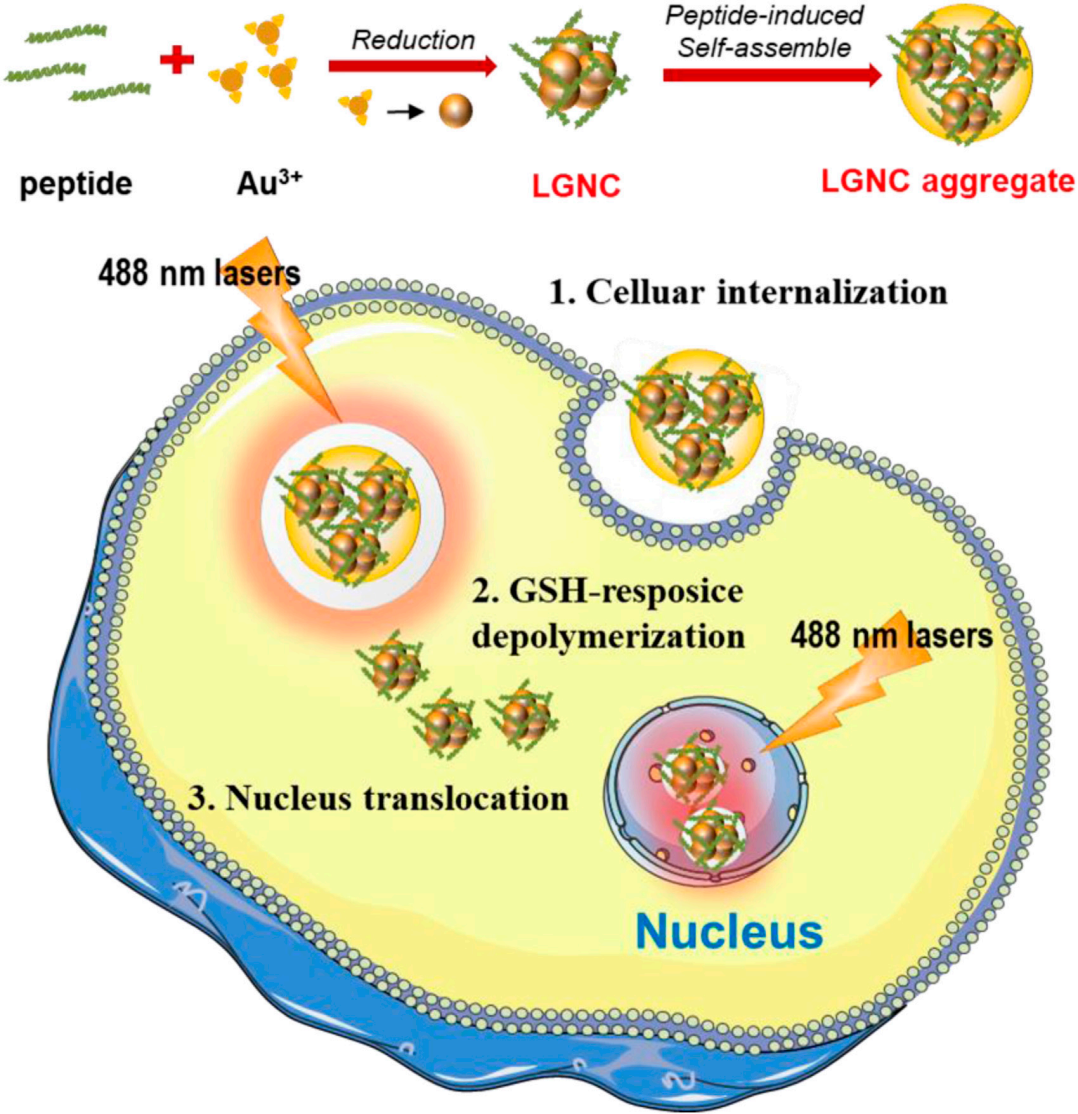
Schematic illustration of lycosin-I-gold nanocluster (GNC) (LGNC) and LGNC aggregate formation and cancer cell nucleus staining after glutathione (GSH)-sensitive depolymerization.

Hence, in this work, we intend to fully utilize the lycosin-I anticancer abilities in constructing fluorescent GNCs for bioimaging of tumor cells. To make it more suitable for GNC preparation, the sequence of lycosin-I is modified as CCY-GGGG-RKGWFKAMKSIAKFIAKEKLKEHL. The modified lycosin-I not only reduced gold ions to atoms but also acted as protective molecular ligands to avoid the further agglomeration of nanoclusters. The introduction of cysteine (C) and tyrosine (Y) residues is essential to construct near-infrared fluorescent GNCs. While C provided -SH for the combination of peptide and Au atoms *via* Au–S bonds, the phenolic hydroxyl groups of Y provided a reducing force for the reaction. The following -GGGG- was a flexible linking domain ensuring the relatively independent space for lycosin-I. The constructed lycosin-I-GNCs (LGNCs) exhibited strong NIR fluorescence, which can be further enhanced by the peptide-induced aggregation. LGNCs also exhibited highly efficient and cell-selective intracellular translocation abilities in several cancer cells like LGNPs. Moreover, the inside nanocomposites could be depolymerized by a high level of glutathione (GSH) leading to nucleus translocation (Figure 1). This present work provides a new choice and strategy for using natural biomolecules in constructing multifunctional nanomaterials for cancer theranostics.

## Materials and Methods

### Materials

GSH, dimethyl sulfoxide (DMSO), CCK-8, Hoechst 33,342, fluorescein isothiocyanate (FITC), and Rhodamine B (Rh B) were purchased from Shenggong Bioengineering (Shanghai) Co., Ltd. Chloroauric acid tetrahydrate HAuCl_4_ 4H_2_O and phosphate buffered saline (PBS) were purchased from Sigma-Aldrich (Shanghai) Trading Co., Ltd., and Abison (Shanghai) Biotechnology Co., Ltd., respectively. DMEM high-glucose medium, fetal bovine serum (FBS), and 0.25% trypsin were purchased from Gibco, United States. Penicillin and streptomycin were purchased from HyClone.

## Methods

### Preparation of LGNC

Lycosin-I was synthesized and purified as we previously described (Wang et al., 2012; Yahia-Ammar et al., 2016; He et al., 2019). In typical GNC synthesis, 5 mg of peptide were dissolved into 258 μl ultrapure water and transferred to a glass bottle after shaking. Then, 516 μl ultrapure water and 30 μl 25 mM HAuCl_4_ 4H_2_O were added and shaken for 2 min. Thereafter, the pH value of the solution was adjusted to 12.5 using NaOH. Then the solution was put still at 50°C for 12 h. After that, the as-prepared LGNCs were concentrated and purified through a 10-kDa ultrafiltration column. The products were stored at 4°C for subsequent use. The concentration of elemental Au in LGNCs was measured by inductively coupled plasma mass spectrometry (ICP-MS, ICAP RQ, Thermo Scientific).

### Characterization of LGNCs

UV-Vis spectra were obtained using a UV-visible spectrometer (UV-2550, SHIMADZU). The fluorescence spectra of LGNCs were measured by a fluorospectrometer (LUMINA, Thermo Scientific). After excitation at different wavelengths, the fluorescence spectra were measured over the 400–850-nm regions, and the excitation spectrum was obtained under fixed emission at the maximum emission peak of 682 nm. A high-resolution transmission electron microscopy (HRTEM, JEM-2100f JEOL) coupled with an energy-dispersive X-ray spectroscopy (EDS) was utilized to collect TEM images and carry out elemental analyses. The size distribution and zeta potential measurements were performed on a dynamic light scatterer (DLS, Zetasizer Nano-ZS90, Malvern).

The QY and fluorescent lifetime were measured by FS 5, Edinburgh Instruments. According the emission peak area and absorbance of LGNC and Rhodamin B, the QY of LGNC could be calculated from Eq. 1 (He et al., 2019):

$$\varphi_{\mathrm{sample}}=\varphi_{\mathrm{ref}}\times \frac{F_{\mathrm{sample}}}{F_{\mathrm{ref}}}\times \frac{A_{\mathrm{ref}}}{A_{\mathrm{sample}}}$$ (1)

### The GSH-Induced Disaggregation

The GSH were introduced to the LGNC solution at final concentrations of 5 and 10 mM. After an 8-h co-incubation, the excess GSH peptides were removed *via* ultrafiltration (10 kD). The morphology and size distribution were detected using TEM and DLS as we mentioned before.

### Cellular Imaging of LGNCs

All the cell lines were purchased from Cellcook Biotech. A549 and Hek293t cells were cultured in DMEM medium supplemented with 15% FBS and 1% penicillin streptomycin at 37°C and 5% carbon dioxide. The 4T1 cells were cultured in RPMI 1640 medium, supplemented with 10% FBS and 1% penicillin streptomycin. Firstly, 10^4^ cells were inoculated in a glass-bottom culture dish and cultured for 24 h before experiments. The cellular internalization was conducted by incubating cells with a final concentration of 1.2 μg/ml LGNCs for 4 h at 37°C and 5% carbon dioxide. Then, cells were carefully washed three times by PBS to remove the excess LGNCs in the medium. Before observing *via* laser scanning confocal microscopy (LSCM, 880, Zeiss), all the cells were stained by DAPI as a reference for nucleus.

The quantitative investments were conducted by flow cytometry (FCM, FACSCalibur, Becton Dickinson) and ICP-MS. The co-incubation conditions were the same as LSCM experiments; then, the cells were harvested, washed, and resuspended after trypsin digestion for FCM tests. In ICP-MS, the harvested cells were completely dissolved by fresh aqua regia. With proper dilution, the Au concentrations in different cells were detected and calculated by ICP-MS.

### Cytotoxicity Tests of LGNCs

The cytotoxicity tests were performed using CCK-8 kits. Briefly, cells were placed into 96-well plates by 5 × 10^3^ cells per well overnight. Then, LGNCs at different concentrations were added to each well. After the 24-h incubation, the previous medium was replaced by a fresh medium containing 10% CCK-8 agents and maintained for 1 h under culture conditions. The absorbance at 450 nm was measured in a microplate reader, and the inhibition of cell growth was calculated by the following formula:

Cell viability (%)=[A (dose) - A(blank)]/[A (control) - a (blank)]  × 100%

## Results and Discussion

### Synthesis and Characterization of LGNCs

**FIGURE 2:**
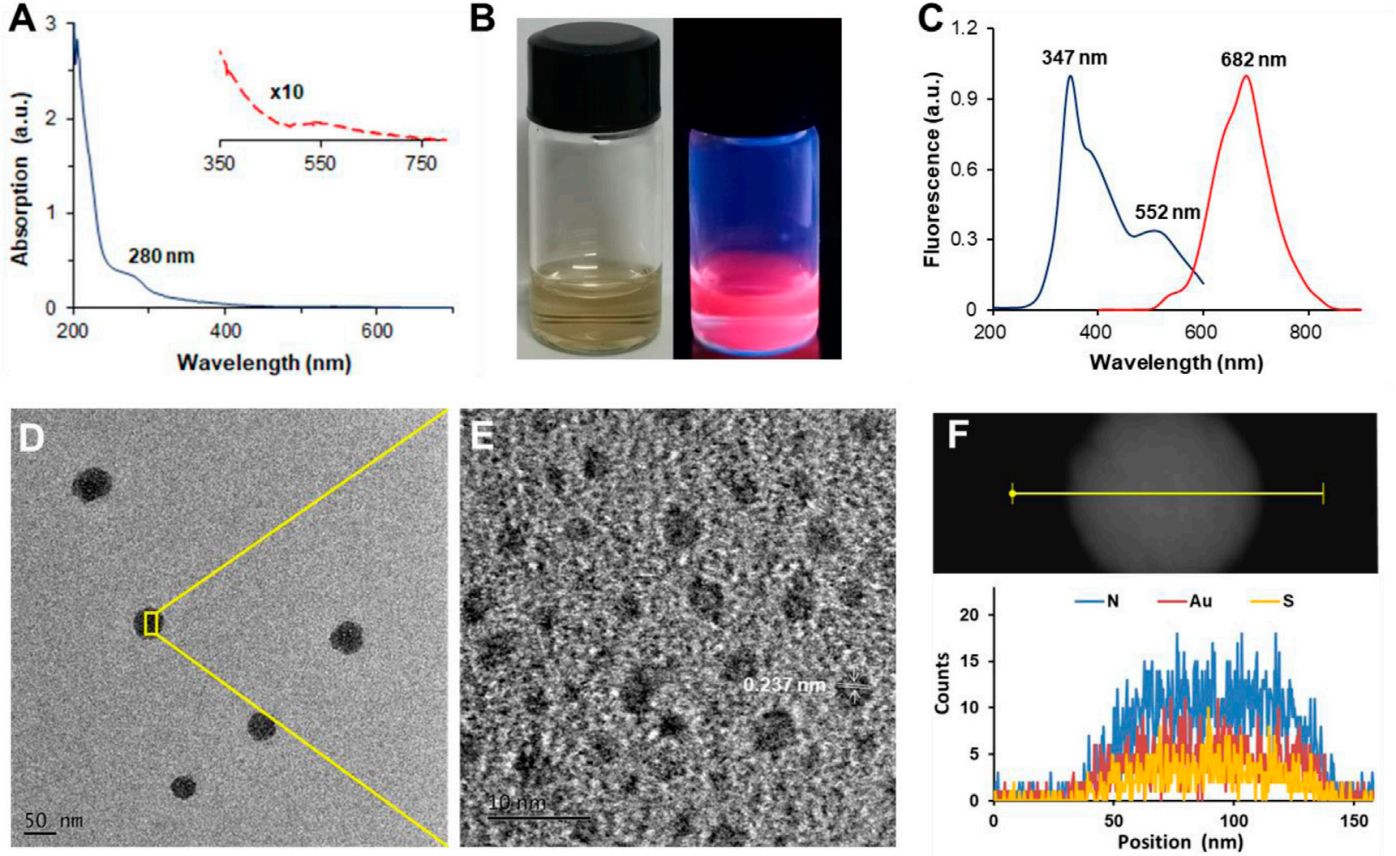
UV-visible spectrum of LGNCs **(A)**. The red dashed line is the enlarged view of 350–750 nm. The photograph of LGNCs under white light (left) and 365-nm hand-held UV lamp excitation (right) **(B)**. Photoemission (red line) and photoexcitation (blue line) spectra of LGNCs **(C)**. High-resolution transmission electron microscopy (HRTEM) images of LGNC aggregates **(D)** and embedded individual LGNC **(E)**. The distribution of elements inside a single LGNC aggregate detected by energy-dispersive X-ray spectroscopy (EDS) **(F)**.

A green “bottom-up” strategy of thiolated GNCs was adopted to prepare LGNCs using CY-lycosin-I as templates and reducing agents simultaneously (Wang et al., 2012; Liang et al., 2017). The UV-vis absorption spectrum of purified LGNCs shows a broad band in the 280–750-nm range (Figure 2A). The small peak at 280 nm can be attributed to the presence of peptide tyrosine residues, which is the typical peak of aromatic amino acid residues in lycosin-I peptide (Supplementary Figure S1). No obvious peak was found at ∼520 nm, which is the typical surface plasmon resonance peak of GNPs, indicating that the as-synthesized nanoclusters were homogeneously formed without gold nanoparticle aggregation (Zheng et al., 2017). Together with a gradient absorption from 350–750 nm detected in LGNCs, the characteristics were similar to the UV-vis absorption spectrum of other reported GNCs (Wang et al., 2012; Liang et al., 2017; Zheng et al., 2017). The transparent LGNC solution is reddish-brown under natural light and exhibits bright near-infrared fluorescence under UV light (Figure 2B). The fluorescence spectrum of LGNCs is shown in Figure 2C. A strong red and NIR emission at the range from 550 to 860 nm was observed under excitation. The maximum excitation and emission wavelengths of LGNCs are 347 and 682 nm, respectively. It was reported that the emission spectra of gold nanoclusters were closely related to templates and pH value of the reaction (Luo et al., 2019; Wu et al., 2020). In this work, as-prepared LGNCs are more favorable for bioimaging, as emission in the NIR region reduces light scattering and tissue spontaneous fluorescence, leading to higher temporal-spatial resolution and deeper imaging depth (Zhu et al., 2020). On the other hand, the PL intensity of LGNCs is also tunable by adjusting the reaction conditions (temperature and time) and especially peptide/Au^3+^ mole ratio (Liang et al., 2017). In the process of optimizing the synthesis conditions, the intensively NIR fluorescent LGNCs could be successfully formed when the reaction temperature reached 50°C and the time exceeded 12 h (Supplementary Figure S2).

Interestingly, with the increasing amount of CY-lycosin-I introduced in the reaction, the fluorescence intensity of LGNCs was significantly enhanced. It is worth noting that lycosin-I is an amphiphilic peptide that could self-assemble into supramolecular nanospheres (40–80 nm) with or without conjugates (Zhang et al., 2017; Zhang et al., 2020). Hence, the enhanced emission of LGNCs with the increasing of peptides may probably be attributed to the peptide-induced aggregation of LGNCs *via* aggregation-induced emission (AIE) and AIE enhancement (AIEE) of thiolate-stabilized GNCs (You and Tseng, 2019; Ran et al., 2019; Zheng et al., 2019; Chen et al., 2020). The TEM images of LGNCs confirmed our hypothesis as displayed in Figures 2D–E. Unlike other typical ultrasmall nanoclusters, the as-synthesized LGNCs formed spherical nanostructures with an average diameter of 53.0 ± 3.5 nm (*n* = 20), and individual nanoclusters with an average size of 2.5 ± 0.7 nm were found inside the LGNC aggregates. The elemental composition of the LGNC aggregates was analyzed by an energy-dispersive spectrometer. Besides C, O, and Cu from the carbon-coated copper grid, the presence of Au, S, and N peaks indicated that the LGNC aggregates were assembled from numerous lycosin-I-protected GNCs (Figure 2F). The quantum confinement effect endows GNCs with molecular-like properties such as discrete electronic transitions leading to photoluminescence, which means that the formation of larger plasmonic GNP brings PL quenching ineluctably. However, the LGNC aggregates exhibited enhanced PL with QY of 9.1% and a prolonged fluorescent lifetime of 2.1 μs compared with other reported GNCs (Supplementary Figure S2) (Wang et al., 2012; Chen et al., 2020). The above results confirmed that the lycosin-I templates on the surface of gold clusters not only protect nanoclusters from forming a larger gold core but also self-assemble to improve the fluorescence of LGNCs *via* the AIEE effect.

### GSH-Induced Depolymerization of LGNC Aggregates

The AIE/AIEE is one of the common strategies for enhancing the luminance of thiolate-protected GNCs for imaging and detection (Ran et al., 2019; Rad et al., 2021). Mechanistically, the restriction of intramolecular vibration and rotation of the ligand layer on the NC surface after aggregation could be the main factors to the PL enhancement of metal nanocluster assemblies, which facilitate the radiative energy transfer *via* restraining ligand-related non-radiative excited state relaxation. A number of well-designed AuNCs have been established based on AIE/AIEE, especially for detection of target analyte with very low concentrations (He et al., 2019; Ran et al., 2019; You and Tseng, 2019; Rad et al., 2021). Among them, GSH is a typical peptide employed in such GNC-based detection systems. It has been reported by different groups that GSH could induce the AIE of several GNCs that used amino acids and micro-molecules as stabilizers such as histidine, arginine, and HS^−^ due to its preference of forming Au–S bonds with GNCs (Zhang et al., 2015; You and Tseng, 2019; Lu et al., 2020).

**FIGURE 3:**
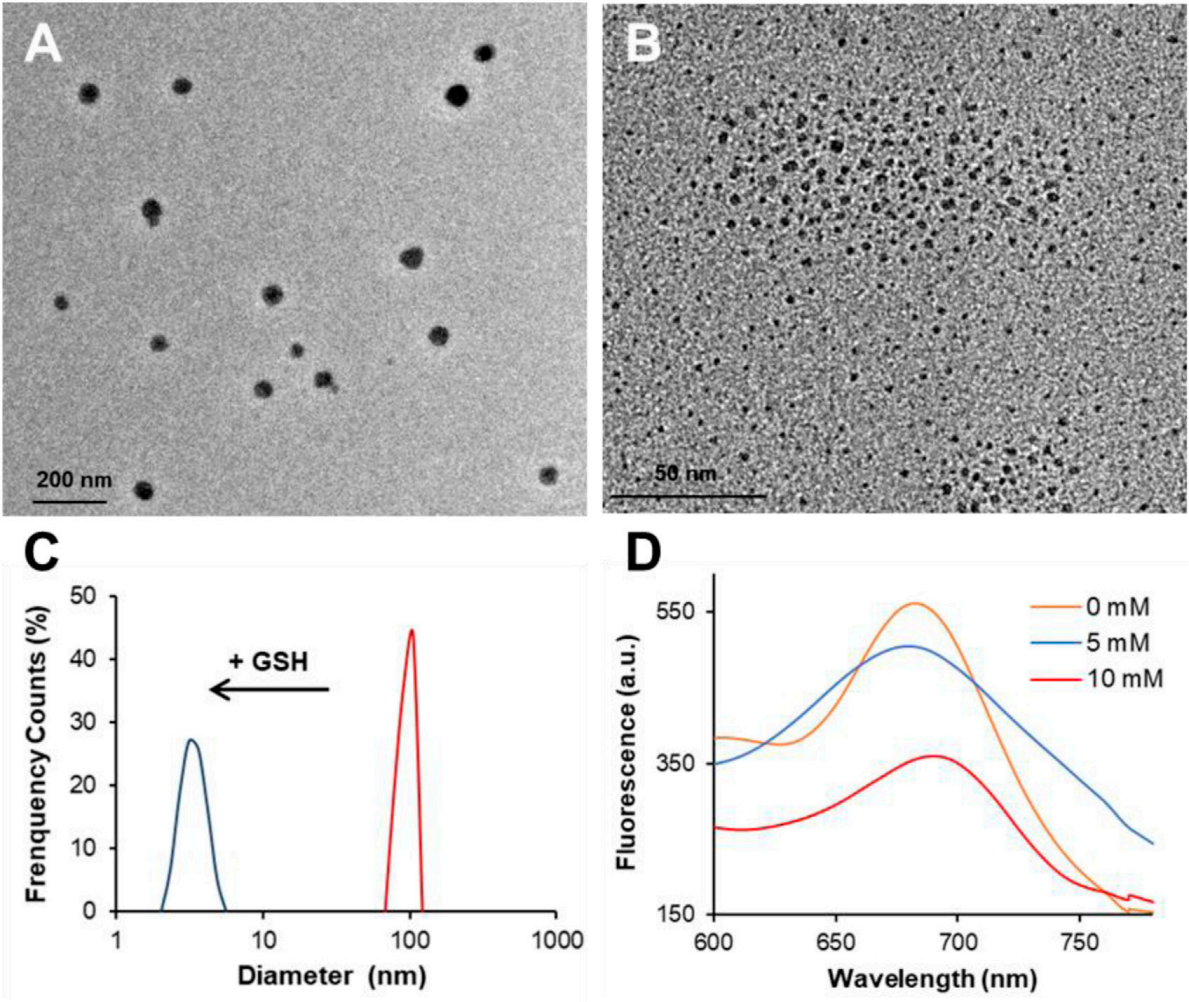
Transmission electron microscopy (TEM) images of LGNC aggregates before **(A)** and after 10-mM GSH treatment **(B)**. The hydrodynamic size variation of LGNCs with and without GSH **(C)**. The fluorescent spectra of LGNCs after GSH treatment at different concentrations **(D)**.

Nevertheless, things could be different in LGNCs considering the NC aggregates have already self-assembled by virtue of conjugated lycosin-I. To investigate that, we introduced excess GSH (with final concentration of 10 mM) to LGNCs and utilized TEM and DLS to explore the changes of peptide–NC assemblies. As shown in Figures 3A,B, LGNC assemblies were spherical in shape with an average size of ∼60 nm, which is coincident with peptide aggregates of lycosin-I as reported previously (Zhang et al., 2017; Zhang et al., 2020). After incubation with 10 mM GSH for 8 h, the assemblies were found depolymerized into individual ultrasmall nanostructures with an average size of ∼3 nm just like the inner NCs of the LGNC aggregates we mentioned in Figure 2. Meanwhile, the same pattern of hydrodynamic size variation has been detected by DLS. The measured hydrodynamic size was about ∼1.8-fold larger than the hydrophobic size observed by TEM due to the shrinking of peptide–NC complex during the drying process on the TEM grid. Likewise, the average size of LGNCs decreased dramatically from 98.8 ± 7.2 nm to 3.4 ± 0.6 nm after co-incubation with GSH (Figure 3C). It suggested that a high concentration of GSH could cause the depolymerization of lycosin-I-induced LGNC assemblies. Meanwhile, a negligible alteration has been detected in LGNC UV-vis spectra at a wavelength range of 450–700 nm after GSH treatment (Supplementary Figure S3). It may be because of the fact that the characteristic of peptide-protected ultrasmall Au clusters, as the chromophores causing UV absorption, remains stable in aggregate or monomer state. Additionally, the zeta potential of tested nanoclusters declined from 13.3 ± 7.3 to 4.8 ± 3.6 mV after GSH treatment. Given that GSH is preferable for forming Au–S bonds due to its negative charges and less steric hindrance than lycosin-I, it is conceivable that the competitive substitution of GSH on the surface of GNCs could cause the disintegration of LGNC aggregates. As to the PL performance of LGNCs, the intensity of GNCs was weakened in a concentration-dependent manner after being treated by GSH. The maximum emission intensity reduced 10.1% at a GSH concentration of 5 mM and 36.0% at 10 mM alongside with the slight alteration of emission spectra of GNCs (Figure 3D). So, that further proved the AIE and AIEE of LGNC aggregates caused by lycosin-I self-assembly can be interrupted by a high dose of GSH, resulting in disaggregation and PL quenching.

### Enhanced and Selective Cellular Internalization of LGNCs

**FIGURE 4:**
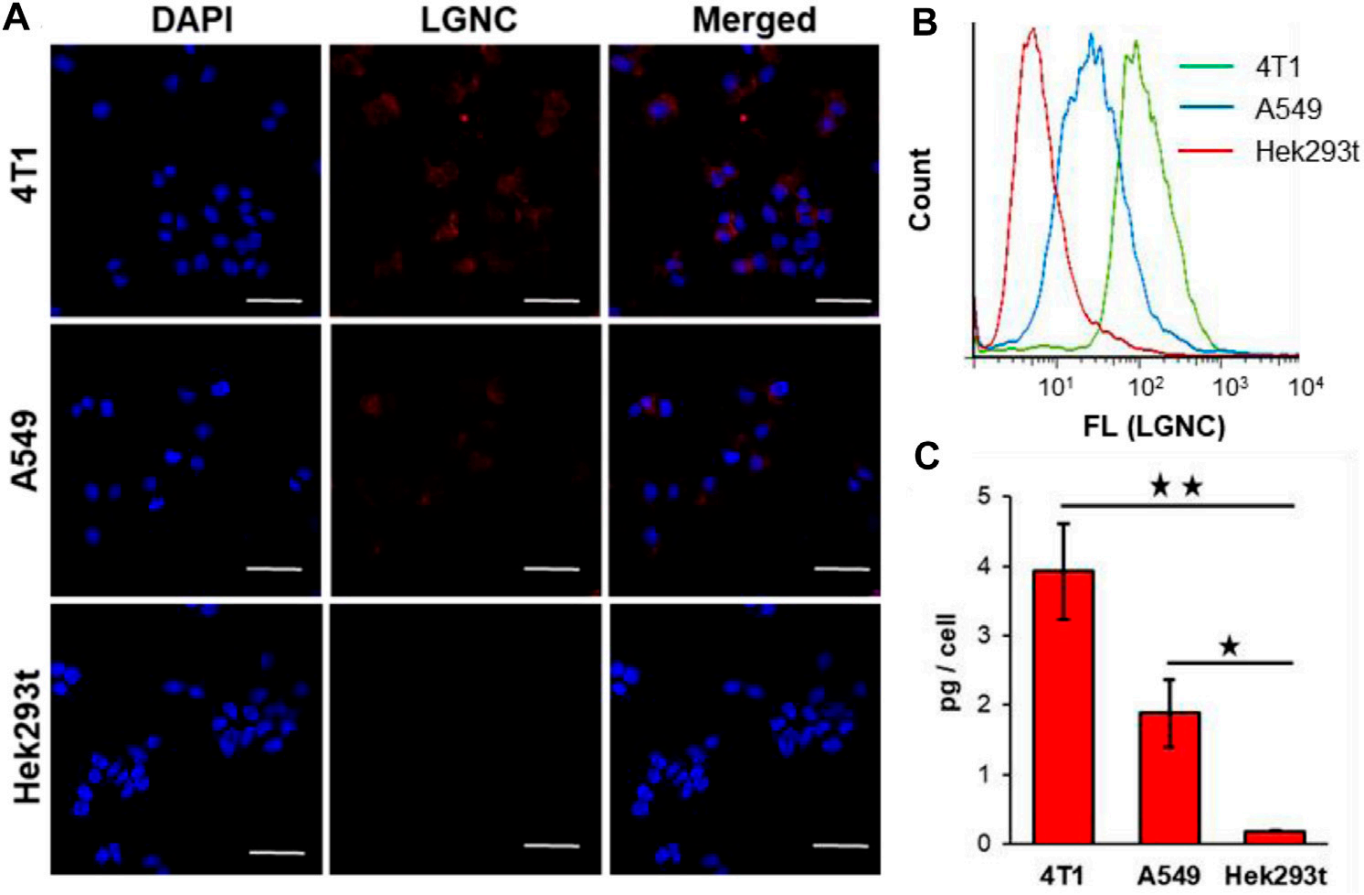
Confocal laser scanning microscopy (CLSM) images of 4T1, A549, and Hek293t cells co-incubated with 1.2 μg/ml LGNCs for 4 h **(A)**. Flow cytometry (FCM) analysis of cellular uptake rates of LGNCs after 4-h treatment **(B)**. Intracellular Au amounts of LGNC co-incubated cells determined by inductively coupled plasma mass spectrometry (ICP-MS) (mean ± SD, *n* = 3) **(C)**. Statistical significance: ^★^ *p*-value <0.05; ^★★^ *p*-value <0.01. The scale bar in CLSM images represents 50 μm.figure 5

Our previous works have revealed the efficient intracellular internalization capacity of lycosin-I and its conjugates with certain cell selectivity (Tan et al., 2017; Zheng et al., 2017). Herein, the cellular uptake of LGNCs was characterized in three different cell lines including two malignant cell lines (4T1 and A549 cells) and one non-malignant cell line (Hek293t cells). In brief, cells after incubation with LGNCs at a final elemental Au concentration of 1.2 μg/ml for 4 h were washed and then observed by laser confocal fluorescence microscopy. As displayed in Figure 4A, the 4T1 cells (murine mammary carcinoma cells) exhibited the most intensive red emitted light (*λ*_em_ = 650 nm) of LGNCs under the excitation of 488-nm laser. While DAPI, which has a blue emission (*λ*_em_ = 440 nm), was used as a reference for nucleus staining; the red emitting signals of LGNCs were found mainly in the cytoplasm domain surrounding the nucleus area. This distinct cellular uptake of LGNCs was similar but slightly attenuated in A549 cells (human non-small cell lung cancer cells). Whereas in non-tumor Hek293t cells (human embryonic kidney cells), the LGNCs seemed to be kept impenetrable in view of the absence of obvious fluorescence detected under identical experimental conditions.

To quantify this cell selectivity of LGNCs, the FCM and ICP-MS analyses were applied in those three cell lines. The FCM histograms of the three cells, monitoring the fluorescence intensity of each cell, are integrated in Figure 4B. Same as the LSCM results, almost all 4T1 cells were stained by LGNCs, while Hek293t cells were similar to control cells without detectable PL signals under 488-nm laser excitation. The highest LGNC uptake rate was detected in 4T1 cells (94.5% according to the FCM scatter diagrams in Supplementary Figure S2), followed by A549 cells (44.7%), and the least was detected in Hek293t cells (6.02%). The accurate Au amounts inside cells were measured and calculated *via* ICP-MS. As shown in Figure 3C, 4T1 cells could uptake 3.9 ± 0.7 pg Au per cell (in total of 40,000 cells) after 4-h incubation with LGNCs, which was nearly 20-fold higher than Hek293t cells (0.2 ± 0.1 pg/cell). Though the Au uptake went down in A549 cells (1.9 ± 0.5 pg/cell), it was still about 10-fold higher than Hek293t cells with statistical difference (*p* < 0.05). The impermeability of LGNCs in human umbilical vein endothelial cells (HUVEC) was similar to that in Hek293t cells (Supplementary Figure S5). The above results confirmed the highly improved and selective intracellular translocation capacity of LGNCs towards tumor cells over non-tumor cells. The heterogeneity of LGNC uptake in different cells may result from the difference in cell membrane surficial electrical properties. In our early works, cationic amphiphilic lycosin-I was found prone to spiral and self-aggregate upon contact with the lipid bilayer on cell membranes (Tan et al., 2016). This dynamic change will be further aggravated when the membrane electronegativity increased, resulting in an intensified interaction between peptides and lipids and an increased membrane permeability. Thereby, in this lycosin-I-protected nanocluster system, where the lycosin-I aggregates have already assembled with GNCs embedded, it is conceivable that the cancerous cells with more negatively charged lipid membrane are more preferable to uptake LGNCs than non-cancerous cells. The above results indicate that lycosin-I template endowed GNCs with not only enhanced cellular internalizationability but also effective selectivity for malignant cells.

### Stepwise Nucleus Staining of LGNCs in 4T1 Cells

**FIGURE 5:**
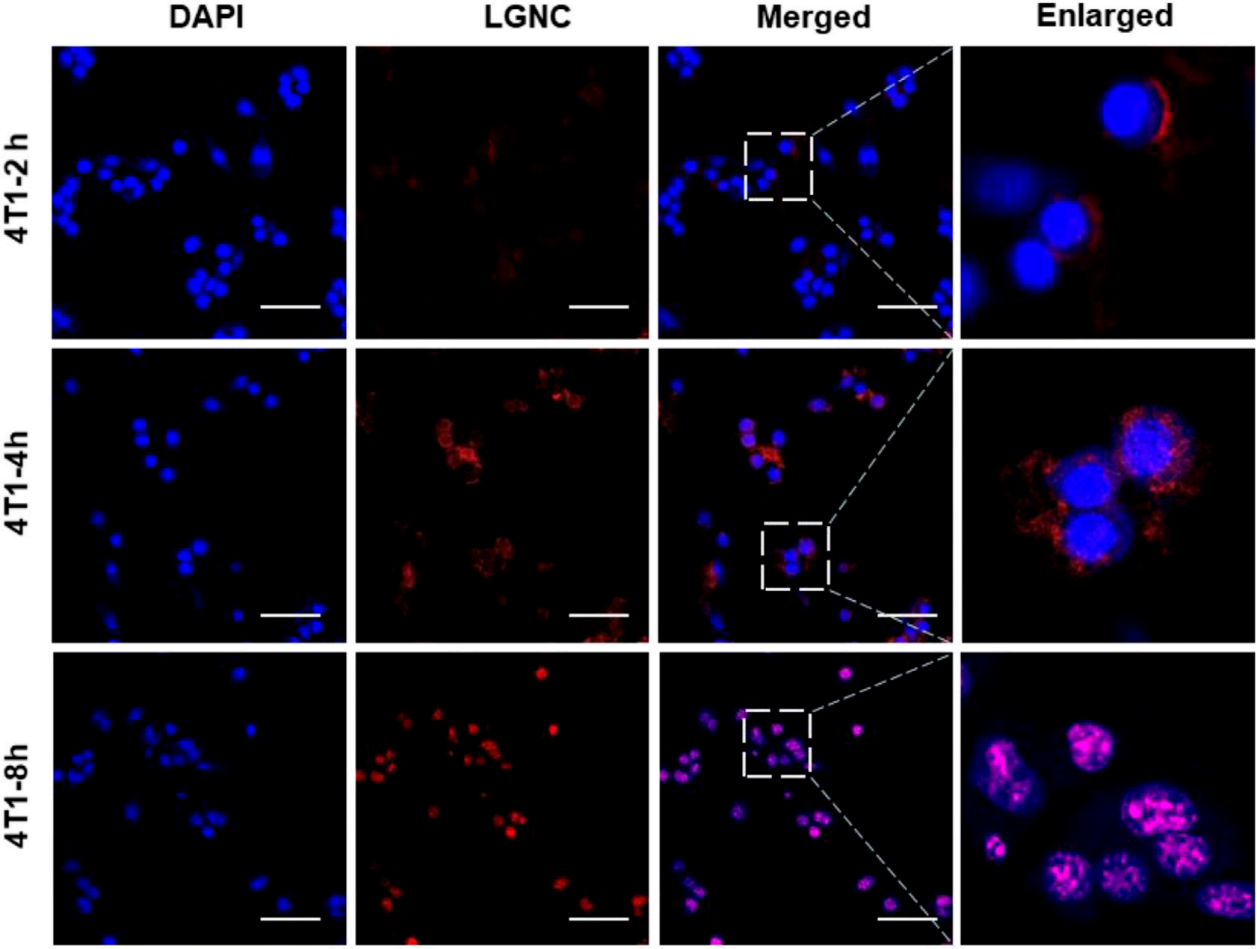
CLSM images of subcellular distribution of LGNCs in 4T1 cells for various incubation times. The scale bar represents 50 μm.

To investigate the subcellular distribution of LGNCs, serial time points of co-incubation were set and the fluorescent images were captured by LSCM at each point in 4T1 cells. In Figure 5, it is clear that LGNCs had already emerged in cells after 2 h of co-incubation, indicating the rapid transmembrane translocation of LGNCs towards 4T1 cells. Same as we mentioned before, the red fluorescent signals inside the cells were enhanced. While LGNCs were found mostly distributed in the cytoplasm, partial overlapping signals of LGNCs and DAPI could be detected in the nuclear areas. The aggregation in the nucleus was intensified with the incubation time prolonging. After 8-h treatment, the co-localization of LGNCs and DAPI was confirmed by the magenta color revealed in the merged images, which means LGNCs were completely located in the cell nucleus. This nucleus-targeted location of LGNCs is similar to other cationic GNCs as reported, such as Tat peptide (a cell-penetrating peptide from AIDS virus) protected GNCs (Wang et al., 2012). However, compared to the fast nuclear staining of Tat-GNCs within 2-h incubation, the subcellular location of LGNCs was stepwisely changed in 8 h with no distinct affection on cell viabilities (Supplementary Figure S6).

We believe that this difference can be ascribed to two reasons. Firstly, it is acknowledged that the nuclear membranes are selectively impermeable except for cargos with nuclear pore complexes (NPCs) targeting ligands and small molecules or particles with a size of less than 8 nm. Therefore, the sub-2-nm Tat-GNCs can enter the cell nucleus and bind to electronegative nucleic acid rapidly, while the intracellular LGNCs are confined to the peptide–GNC aggregates in the cytoplasm. The other key is the higher GSH level in 4T1 cells. The high level of GSH in cancer cells is one of the most important physiological parameters of tumor microenvironment (Wu et al., 2004; Zhang et al., 2018). Given that the LGNC assemblies could be disaggregated into ultrasmall nanoclusters by GSH as we proved, it is reasonable that the high level of GSH in cancer cells is the major triggering factor for the redistribution of LGNCs inside 4T1 cells.

## Conclusion

In conclusion, in this work, we employed the natural anticancer peptide lycosin-I for the construction of NIR fluorescent gold nanoclusters *via* one-pot reaction achieving the integration of synthesis and functionalization. The as-prepared LGNCs exhibited excellent PL properties, which can be further enhanced by the peptide-induced NC aggregation. The LGNC aggregates possess highly efficient and cell-selective intracellular translocation abilities in several cancer cells we tested. The intracellular LGNC aggregates can be depolymerized into individual LGNCs triggered by the high GSH level of tumor cell microenvironment and realize the nuclear targeting translocation in cancer cells. These results provide a new choice and strategy for specific cancer cell nuclei imaging and delivery benefiting the theranostics for malignant tumor.

## Data Availability

The original contributions presented in the study are included in the article/Supplementary Material, further inquiries can be directed to the corresponding author.
