# Supplementary material for: Spider Toxin Peptide-Induced NIR Gold Nanocluster Fabrication for GSH-Responsive Cancer Cell Imaging and Nuclei Translocation
Source: Front Bioeng Biotechnol. 2021 Nov 16;9:780223. doi: 10.3389/fbioe.2021.780223 (PMC8635238; doi:10.3389/fbioe.2021.780223)
Supplement: Supplementary file 1 [file DataSheet1.docx]

***Supporting Information***

Huaxin Tan^a^, Sisi Liu^b^, Yaolin He^c^, Guofeng Cheng^a^, Yu Zhang^a^, Xiaojie Wei^d^, and Lidan Hu^a,^ *

1. Department of Biochemistry and Molecular Biology, the Key Laboratory of Ecological Environment and Critical Human Diseases Prevention of Hunan Province Department of Education, School of Basic Medicine, Hengyang Medical School, University of South China, Hengyang 421001, P. R. China.
2. School of Public health, Hengyang Medical School, University of South China, Hengyang 421001, P. R. China.
3. The Second Affiliated Hospital, Department of Radiotherapy, Hengyang Medical School, University of South China, Hengyang 421001, P. R. China.
4. School of Pharmacy, Hengyang Medical School, University of South China, Hengyang 421001, P. R. China.


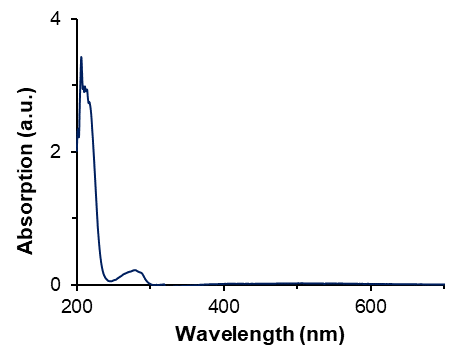


**Figure S1**. UV-vis spectrum of lycosin-I peptide.


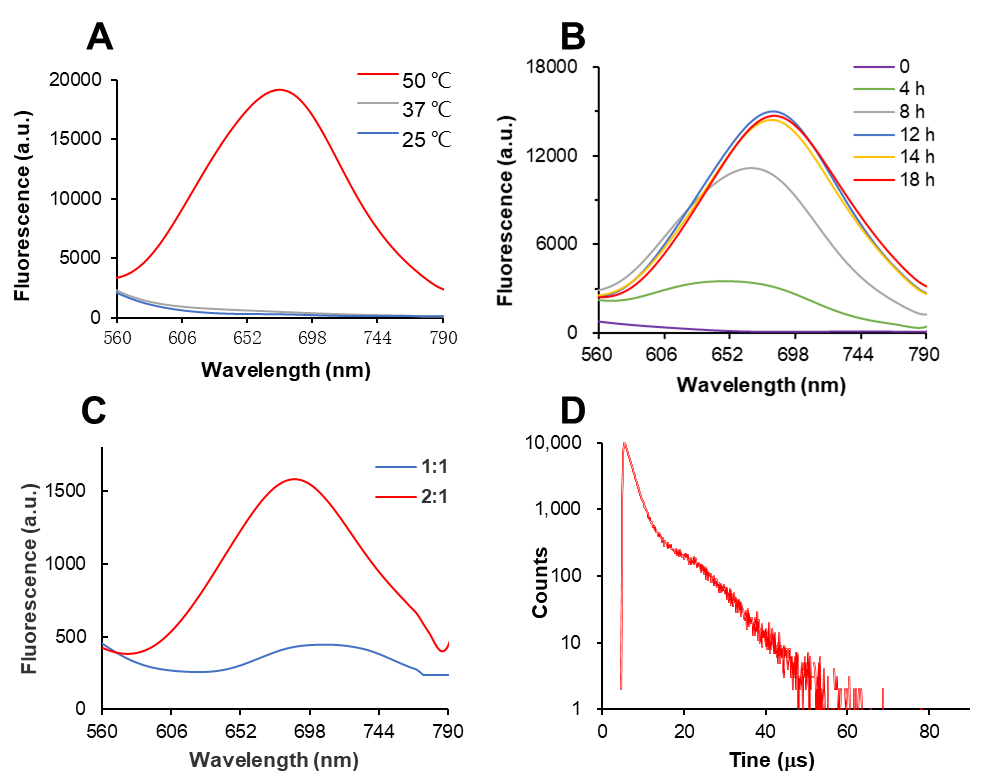


**Figure S2**. Fluorescent spectra of LGNCs under different synthesis condition of temperature (A), reaction time (B) and peptide-Au^3+^ ratio (C). Fluorescence decays for LGNCs in aqueous solution (D).


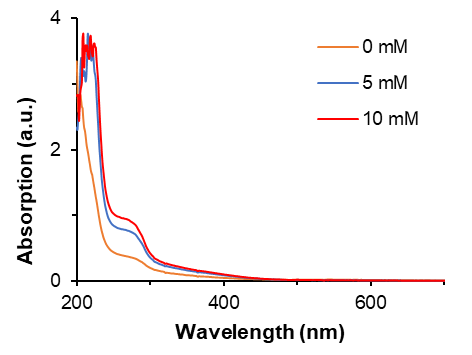


**Figure S3**. UV-vis spectrum of after GSH treatment at different concentration.


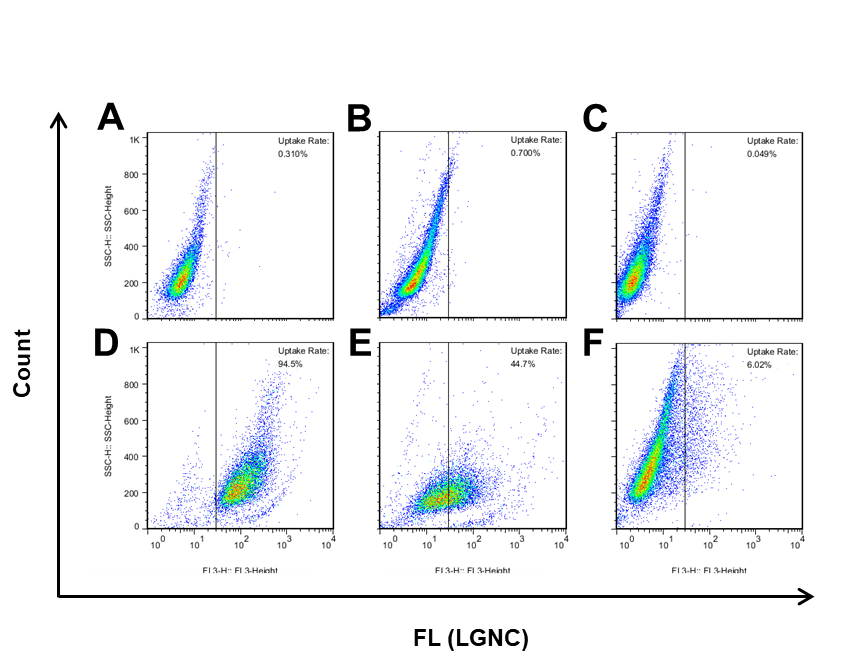


**Figure S4**. FCM scatter diagrams of cells with PBS treatment and LGNC co-incubation in 4T1 (A, D), A549 (B, E) and Hek293t (C, F) cells.

**Figure S5**. CLSM images of HUVEC co-incubated with 1.2 μg/mL LGNCs for 4 hours (A). The scale bar in CLSM images represents 50 μm. FCM scatter diagrams of cells with PBS treatment and LGNC co-incubation in HUVEC (B).


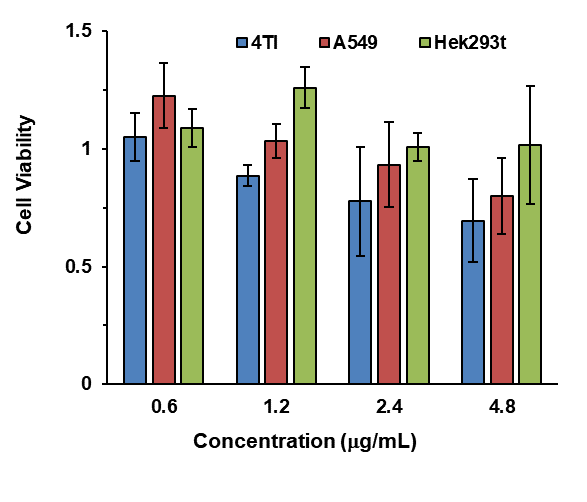


**Figure S6**. Cell viability of 3 cells after 24-hour co-incubation with LGNCs at different concentrations (mean±SD, n=3).
